# Local tourism experiences at World Heritage rice terrace sites in China: A comparative study of Hani Terraces and Longji Terraces

**DOI:** 10.1371/journal.pone.0349872

**Published:** 2026-06-01

**Authors:** Zhen Yang, Ming Liu, Longyi Cai

**Affiliations:** 1 College of Design and Innovation, Fujian Jiangxia University, Fuzhou, China; 2 Faculty of Tourism and Community Development, Kokugakuin University, Kanagawa, Japan; 3 College of Architecture and Civil Engineering, Xiamen Institute of Technology, Xiamen, China; Sultan Qaboos University, OMAN

## Abstract

Rice terraces, as quintessential cultural landscapes shaped by long-term human-land interactions, embody productive, ecological, and cultural value while evolving into significant rural tourism destinations. This study examines two World Heritage–listed sites in China—Hani Terraces in Yunnan Province and Longji Terraces in Guangxi Province—using 4,018 visitor reviews collected from the Ctrip platform. Word frequency analysis was conducted to identify recurrent terms in visitor comments and to determine key dimensions of tourism experience. Semantic co-occurrence network analysis was then applied to explore structural relationships among core terms and to reveal differences in visitor perceptions. In addition, chi-square tests were used to identify statistically significant differences in evaluation characteristics and to examine patterns of negative feedback. The results show that Longji Terraces benefit from higher tourism visibility and a larger volume of online reviews, whereas visitors to Hani Terraces place greater emphasis on cultural and heritage values. In contrast, visitors to Longji Terraces tend to focus more on leisure-oriented tourism services. Negative feedback for both sites primarily centers on transportation and management issues. Weather conditions significantly impacted sightseeing at Hani Terraces, while Longji Terraces faced complaints mainly related to service quality. Overall, this study highlights the heterogeneity of tourism experiences across terraced heritage landscapes and identifies key factors shaping visitor perceptions. By extending the application of user-generated content (UGC) in agricultural heritage tourism research, the findings provide empirical evidence to support tourism management, landscape conservation, and the sustainable development of agricultural heritage sites.

## 1. Introduction

Rice terraces, as products of long-term human-nature interaction, constitute a composite landscape system integrating production, ecological, and cultural functions [1,2]. Their spatial morphology not only reflects profound human understanding of and adaptive strategies toward the natural environment but also embodies the organic fusion of human ingenuity and natural processes [3,4]. As natural conditions and social contexts have continued to evolve, terraced landscapes have played a vital role in shaping regional landscape patterns while consistently supporting agricultural ecosystem stability and expressing distinctive aesthetic values [5–7]. In 1995, the inscription of the Rice Terraces of the Philippine Cordilleras as the world’s first agricultural cultural landscape on the UNESCO World Heritage List marked a turning point in international scholarly engagement with terraced landscapes. Since then, terraces have emerged as a prominent research focus within the global field of agricultural heritage studies [8].

However, due to socioeconomic and environmental factors, the abandonment and fallow of terraced fields have become increasingly pronounced [9–11]. As traditional rice-farming culture gradually weakens [12], the terraced landscape ecosystem also faces severe challenges [13]. In recent years, villages centered around terraced fields and characterized by mountainous agricultural landscapes have gradually emerged as key destinations for rural tourism, owing to their unique topography, long-standing rice-farming systems, and rich traditions of folk culture. The development of rural tourism has partially mitigated the trend of continuous abandonment in some terraced fields, injecting new momentum into the diversified transformation and sustainable development of these villages [14]. Some terraced fields have been transformed into comprehensive scenic areas integrating ecotourism and cultural experiences, reflecting a deep coupling between traditional agricultural landscapes and modern tourism consumption demands. Against this background, understanding how visitors perceive, experience, and evaluate terraced cultural landscapes has become particularly important, as visitor perceptions directly shape tourism development patterns and, at a deeper level, influence the long-term conservation and sustainable use of terraced landscapes.

From the perspective of landscape perception theory, tourists’ evaluations of terraced field tourism not only reflect their subjective understanding of landscape integrity, cultural authenticity, facility suitability, and local atmosphere, but also embody the cognitive judgments and emotional responses formed during the tourism experience. These perceptions contribute to the ongoing reinterpretation of heritage values and the spatial reproduction of terraced heritage sites. Drawing on destination image theory and the cognitive–affective–conative framework, tourists’ perceptions directly influence behavioral intentions, consumption decisions, and patterns of social media sharing [15,16]. Through processes of evaluation and feedback, such perceptions further shape local governance approaches to resource allocation, cultural interpretation, and landscape management within tourism planning [17]. Therefore, systematically collecting and analyzing visitors’ evaluations, preferences, and needs has become a critical component in the tourism development and landscape conservation efforts of terraced heritage sites.

As a traditional rice-growing nation, China features extensive rice terraces across its southern regions. Their orderly, layered spatial patterns have intertwined with indigenous rice cultivation cultures, forming agricultural landscapes with distinctive regional characteristics. Among these, Hani Terraces in Yunnan and Longji Terraces in Guangxi have been inscribed on the World Heritage List for their outstanding landscape value and the significance of their traditional agricultural systems. Since their heritage recognition, both sites have gradually developed into representative rice terrace tourism destinations, with visitor numbers showing an overall steady growth trend. With the continued expansion of tourism in terraced villages, tourists’ immediate perceptions and subjective experiences are increasingly recorded and disseminated through social media platforms in the form of user-generated content (UGC), including textual reviews, images, and videos. This UGC-based perceptual information not only provides a crucial perspective for revealing visitors’ recognition of the recreational, aesthetic, and cultural values of the terraced landscapes but also offers empirical support and a data foundation for evaluating the effectiveness of landscape conservation and optimizing management strategies at terraced heritage sites.

Against this backdrop, this study systematically examines tourist perceptions, evaluations, and experiential characteristics of the tourism environments at Hani Terraces and Longji Terraces using visitor review data generated on social media platforms. The research aims to address the following core questions: (1) What differences exist in the overall tourism experiences of the Hani Terraces and the Longji Terraces? (2) How do tourists’ perceptual differences between the two sites manifest, particularly in terms of experiential focus and semantic thematic structures? (3) What differences characterize the types, thematic emphases, and emotional tendencies of negative evaluations at the two sites, and what underlying factors may explain these patterns? Building upon these questions, this study seeks to provide theoretical references and practical insights for tourism management, landscape conservation, and sustainable development in terraced heritage sites and other mountainous rural areas.

## 2. Literature review

### 2.1. Rural tourism through the lens of social media

Compared to traditional research methods such as questionnaires [18] and interviews [19], UGC-based analysis offers new avenues for studying tourism experiences [20], destination image perceptions [21], and tourism behavior patterns [22]. UGC data boasts advantages such as diverse sources, timely updates, and large volumes. It is typically accompanied by explicit temporal information and geographic location tags [23,24], enabling researchers to track the authentic behavioral trajectories and subjective cognitive processes of individuals or groups across specific spatial and temporal scales. For potential travelers, reviews, guides, and experiences shared by actual tourists are often perceived as more authentic and credible, significantly influencing their travel intentions and destination choices [25]. Meanwhile, UGC data provides decision-making references for tourism management authorities and destination operators [26].

Existing studies from a social media perspective have examined themes such as national tourism image building [27–30], analyses of tourists’ attitudes toward urban cultural heritage sites [31,32], and explorations of experiences in natural scenic areas [33–35] or coastal landscapes [36–38]. In contrast, UGC-based research on rural tourism remains relatively underdeveloped. Current studies have mainly focused on analyses of tourism demand preferences, conceptualizing rural tourism experiences and identifying their key elements [39], and examining visitors’ perceptions and expressions of village natural environments [40]. Additionally, relevant studies have examined the role of UGC in destination branding, visitor engagement, tourism revitalization [41], and stimulating revisit intentions [42]. Based on this, this study adopts a visitor experience perspective and seeks to further enrich and expand rural tourism research from a social media viewpoint.

### 2.2. Tourism perceptions of terraced heritage sites through the lens of social media

A limited number of studies have attempted to analyze perceptions and emotional experiences of terraced field tourism based on UGC. Drawing on digital materials such as social media, travel review websites, and visitor photography, these studies combine methods including text analysis, affective computing, and visual recognition to extract perceptual characteristics and emotional tendencies from unstructured data. Some of this work focuses on single terraced heritage sites, demonstrating strong regional specificity [43,44]. Other studies adopt a multi-case perspective, thereby extending the macro-level scope of research on terraced tourism perception [45,46]. Overall, however, existing research remains relatively limited in terms of cross-regional feature comparison and analyses of geographical heterogeneity, making it difficult to fully capture the uniqueness of different terraced heritage sites and the sources of variation.

## 3. Materials and methods

### 3.1. Research scope

This study selected two representative terraced heritage sites in China—Hani Terraces in Honghe, Yunnan and Longji Terraces in Guangxi—as subjects for comparative analysis. The selection of research subjects was primarily based on the following three considerations:

First, regarding the volume and diversity of UGC data and reviews, this study conducted preliminary data collation on China’s eight most representative terraced heritage sites (Table 1), including Hani Terraces in Yunnan, Longji Terraces in Guangxi, the Yunhe Terraces in Zhejiang, the Ziquejie Terraces in Hunan, the Jiabang Terraces in Guizhou, the Shangbao Terraces in Jiangxi, the Fengyan Terraces in Shaanxi, and the Lianhe Terraces in Fujian. Statistical results indicate that only Hani Terraces in Yunnan and Longji Terraces in Guangxi have reached a scale of thousands of valid reviews (Ctrip data) which cover multiple dimensions such as landscape perception, tourism experience, and cultural impressions, demonstrating high research representativeness and robust data support.

**Table 1:** Number of Ctrip reviews for representative terraced heritage sites in China.

Terraces	County	Province	Major Ethnic Groups	Heritage Type	Total Ctrip Reviews	Ctrip Reviews Collection	Valid Ctrip Reviews
**Hani Terraces**	Yuanyang	Yunnan	Hani	2010 GIAHS	2,036	1,341	**1,303**
				2013 WCH			
				2013 China-NIAHS			
**Longji Terraces**	Longsheng	Guangxi	Zhuang, Yao, Miao, Dong	2014 China-NIAHS	6,168	2,977	**2,715**
				2018 GIAHS			
Yunhe Terraces	Yunhe	Zhejiang	She	2015 China-NIAHS	5,368	2,992	(782)
Ziquejie Terraces	Xinhua	Hunan	Miao, Yao, Dong, Han	2013 China-NIAHS	378	218	
				2018 GIAHS			
Jiabang Terraces	Congjiang	Guizhou	Miao	2011 GIAHS	347	192	
Shangbao Terraces	Chongyi	Jiangxi	Han (Hakka)	2014 China-NIAHS	120	116	
				2018 GIAHS			
Fengyan Terraces	Hanyin	Shaanxi	Han	2021 China-NIAHS	59	25	
Lianhe Terraces	Youxi	Fujian	Han	2013 China-NIAHS	26	15	
				2018 GIAHS			
Total					14,502	7,876	**4,018**

Second, both sites possess outstanding heritage value and abundant cultural tourism resources, with the characteristic of advancing heritage conservation and tourism development in tandem. Both terraced landscapes feature large-scale and contiguous rice terraces of significant heritage value. The predominantly ethnic minority populations create a unique cultural atmosphere, where ethnic festivals and agricultural activities complement each other, forming a distinct cultural tourism attraction.

Third, in terms of academic research, both Hani Terraces and Longji Terraces are frequently cited case studies in existing research. The accumulated findings from these studies provide crucial theoretical support and comparative references for this research. In summary, selecting these two sites as the core subjects of this study ensures both sufficient data scale and research depth, while also offering typicality and broader applicability.

**Fig 1:**
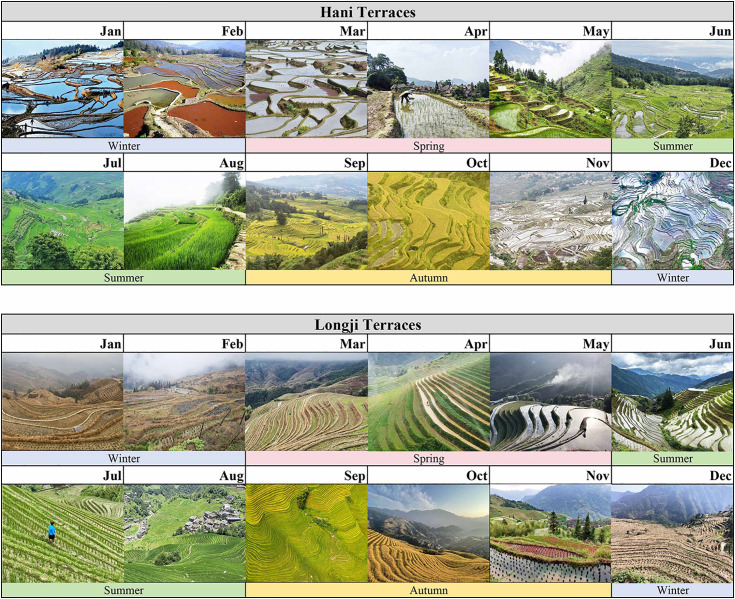
Four Seasons of Terraced Fields.

Hani Terraces are located in Yuanyang County, Honghe Hani and Yi Autonomous Prefecture, Yunnan Province, and have a history of rice terrace cultivation spanning over 1,300 years. The ancestors of the Hani people constructed terraces in mountainous gorges, leveraging the terrain and water sources to sustain their livelihoods through rice farming. In 2010, the site was designated as a Globally Important Agricultural Heritage System (GIAHS). In 2013, it was inscribed on the UNESCO World Heritage List under the name “Cultural Landscape of Honghe Hani Rice Terraces” [47]. The heritage area spans 166.03 km², with its core zone comprising four major terraced fields: Jingkou, Bada, Duoyishu, and Laohuzui. As shown in Fig 1, Hani Terraces undergo irrigation from November to March, followed by rice transplanting in April and harvesting in October. The irrigation period offers the optimal viewing window, when the water surfaces mirror the sky, clouds, and mountains, creating a landscape of exceptional aesthetic value. Following its successful inscription on the UNESCO World Heritage List in 2013, the local tourism industry experienced rapid growth, with both visitor numbers and tourism revenue increasing steadily [48]. Given the central role of the Hani Terraces within the county-level tourism system, this study adopts tourism statistics at the Yuanyang County level to provide a macro-level reflection of overall tourism development trends associated with the Hani Terraces (Table 2). At the same time, tourism expansion has also brought multiple challenges, including terraced field conservation, cultural preservation, and rural governance [49].

**Table 2:** Tourist Arrivals and Tourism Revenue Statistics.

Year	2012	2013	2014	2015	2016	2017	2018	2019	2020	2021	2022	2023	2024
**Yuanyang County (Hani Terraces)**													
Tourist arrivals (10,000 persons)	94.15	107.38	125.30	156.50	220.60	332.09	387.51	430.00	230.29	278.80	419.08	428.93	463.87
Tourism revenue (billion CNY)	10.40	13.15	17.60	19.88	30.28	51.40	67.20	79.44	32.34	38.91	45.48	53.62	56.71
**Longsheng County (Longji Terraces)**													
Tourist arrivals (10,000 persons)	224.37	247.90	273.50	553.45	647.38	777.36	860.03	995.40	698.30	803.03	704.68	1011.13	1121.67
Tourism revenue (billion CNY)	19.04	24.09	30.32	46.16	66.50	83.38	105.08	137.79	83.52	102.40	85.25	125.88	138.25

Data sources: Government work reports of Yuanyang County People’s Government (2012–2024); Midterm evaluation report of the 12th Five-year plan of Longsheng County (2012); Investment guide for Longsheng County (2013); Statistical communiqué on the national economic and Social Development of Longsheng County (2014–2024).

Longji Terraces are located in Longsheng County, Guilin City, Guangxi Zhuang Autonomous Region, with an agricultural history dating back more than 2,300 years [50]. Distributed across steep mountainous terrain, they have been cultivated and maintained for generations by four ethnic minority groups: the Zhuang, Yao, Miao, and Dong peoples. In 2018, as an important component of the “Southern Mountainous Rice Terrace System of China”, Longji Terraces were recognized by the Food and Agriculture Organization of the United Nations as a Globally Important Agricultural Heritage System (GIAHS). The scenic area covers approximately 66 km² and includes three major terraced field viewing zones (Dazhai, Ping’anzhai, Guzhuangzhai) and the Huangluo Hongyao ethnic village. As shown in Fig 1, Longji Terraces undergo water filling from April to May, rice transplanting in June, and harvesting in October. Unlike the flooded landscapes of Hani Terraces, Longji Terraces exhibit particularly distinctive autumn scenery. During harvest season, golden rice fields cascade across the terraces, creating visually stunning waves of gold that attract large numbers of visitors. After years of tourism development, the Longji Terraces have become one of the key industries driving economic growth in Longsheng County. Given their pivotal role in county-level tourism development, this study likewise adopts tourism statistics at the Longsheng County level to describe the long-term trends in tourism development associated with the Longji Terraces (Table 2). At the same time, while tourism has significantly boosted the local economy, labor outflow and inadequate terrace maintenance have become increasingly prominent issues, posing challenges to the landscape’s sustainability.

### 3.2. Data source

The UGC data utilized in this study primarily originates from Ctrip, a renowned Chinese online travel service platform. As China’s leading comprehensive tourism service platform [51], Ctrip boasts a vast user base with high activity levels, accumulating a substantial and continuously growing volume of travel and attraction review data [52]. Its review data not only provides crucial travel reference information for potential tourists but also offers vital data support for various tourism-related research [53].

On 24 June 2025, user review data for eight terraced heritage sites were collected from Ctrip. On the day of data collection, the platform displayed a cumulative total of 14,502 historical reviews. Using the Octoparse web crawler, the research team extracted all reviews that could be loaded through the web interface. Due to limitations related to the platform’s loading mechanisms and access permissions, a total of 7,876 reviews were ultimately obtained (Table 1). After manual data cleaning, this study selected the Honghe Hani Terraces in Yunnan and the Longji Terraces in Guangxi, each with more than 1,000 valid reviews, as the core research cases.

During data processing, it was found that the earliest retrievable reviews for both sites dated back to 2016; however, review data for the entire year of 2017 were missing. From 2018 onward, the volume of reviews became relatively complete. Accordingly, this study primarily selected user reviews posted from 2018 onward as the analytical sample. In principle, the complete review records obtained via the Octoparse crawler should include fields such as user ID, rating, review text, posting date, and reviewer location. It should be noted that Ctrip only began displaying reviewer location information in July 2022; reviews posted prior to that time were therefore mostly marked as having unknown locations. Given the incompleteness of this information, reviewer location was not included in the analysis.

In addition, some retrieved reviews lacked posting dates. To address this issue, the research team manually cross-checked each case against the original review webpages to verify and supplement missing information. Reviews for which complete content or posting dates could not be recovered were excluded from the dataset. Meanwhile, duplicate reviews were automatically identified and removed using the built-in deduplication function of the Octoparse crawler. During the data cleaning process, no clear evidence of spam, bot-generated, or promotional content was identified in the review data for Hani Terraces and Longji Terraces. It should be noted that a small number of reviews show discrepancies between the posting date and the travel time mentioned in the review text. Given their limited number and random distribution, this issue is unlikely to have a substantial impact on the overall results, but it is still regarded as a potential limitation related to metadata consistency in this study.

Data cleaning and organization were completed in late July 2025. Building on the data collected in June, newly posted reviews from July were incorporated, ultimately forming the core dataset for analysis. This dataset comprised 1,303 reviews for Hani Terraces (January 2018–July 2025) and 2,715 reviews for Longji Terraces (January 2018–July 2025). All downloaded data were organized in Excel format. Prior to analysis, the review texts underwent preprocessing, including emoji transcoding, punctuation and word segmentation standardization, correction of Chinese spelling and grammatical errors, and removal of irrelevant symbols, to ensure the accuracy and operability of textual data during sentiment and content analysis. Meanwhile, all data used in this study were obtained from publicly accessible user review pages and consisted exclusively of anonymized textual content. No personally identifiable information was involved, and all analyses were conducted at an aggregate level without any interaction with the individuals who generated the data. Therefore, formal ethics approval and informed consent were not required. In addition, all data collection and analysis procedures complied with the terms and conditions of the Octoparse web crawler, and only publicly accessible user review content was collected.

### 3.3. Research methods

#### 3.3.1. Overall comment data and word frequency analysis.

**Fig 2:**
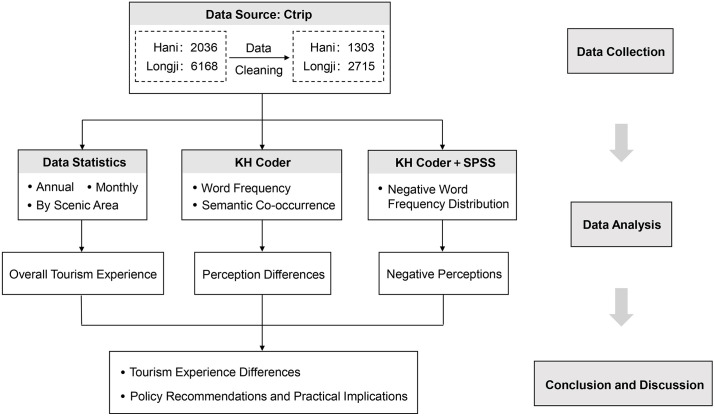
Research framework.

As shown in Fig 2, the study first categorized the comments according to their posting time and ratings using Excel spreadsheets to observe the distribution characteristics of comment numbers and scores across different years for both terraces, and further compare inter-annual variations. Given the distinct seasonal characteristics of terraced landscapes, this study also organized comment content at the monthly level. Average ratings were calculated for each month to examine the monthly patterns of comment volume and rating distribution, thereby providing methodological support for examining the influence of seasonal factors on overall tourism experiences. In addition, review data were aggregated at the attraction level to compare the number of comments and the structure of positive and negative evaluations across different scenic spots. This approach enabled an analysis of spatial differences in visitor engagement and evaluation patterns within the terraced heritage sites. Overall, these analyses aim to reveal the general characteristics of tourism experiences at the two terraced heritage sites.

The study employed KH Coder 3 for word frequency analysis of visitor comments. Developed by Japanese scholar Koichi Higuchi, KH Coder 3 possesses multilingual text analysis capabilities, supporting research in Japanese, Chinese, English, French, and other languages [54]. The original Chinese-language attributes of the review texts were retained, and KH Coder 3 was used to perform word segmentation and word frequency analysis. Words were filtered by part of speech, including nouns, proper nouns, tags, adjectives, adjectives (Penn Treebank tag: JJ), adverbs, and verbs. Considering that visitor experiences are primarily reflected through nouns indicating objects of attention and adjectives conveying emotional attitudes and evaluative tendencies, this study designated nouns (including proper nouns and tags), adjectives (including Penn Treebank tag: JJ) as core analytical categories. To further enhance analytical consistency and accuracy, the extracted terms were subsequently subjected to synonym merging to reduce semantic fragmentation (S1 Appendix). Meanwhile, during the word segmentation stage, multiword expressions referring to scenic area names and core terms closely related to the research theme were predefined to prevent them from being split during tokenization and were treated as single semantic units.

Finally, the extracted vocabulary was systematically categorized into five core themes, i.e., “Terraced Landscape”, “Season/Time/Climate”, “Culture and Heritage”, “Human Factors”, and “Services”. “Terraced Landscape” is the core object of visitor perception. Its significance extends beyond the singular element of “Terraced Fields”, encompassing the integrated landscape formed by surrounding natural environments such as villages, water systems, and forests related to rice cultivation. “Season/Time/Climate” directly impacts the visual appeal and presentation characteristics of the terraced landscape, thereby significantly influencing the tourism experience. “Culture and Heritage” reflects the historical and cultural value embodied by the terraces as agricultural heritage sites. “Human Factors” encompass visitor group composition and interactions with local residents. “Services” represent tourism infrastructure and management standards.

Existing research on destination image, tourism experience, and heritage tourism suggests that tourists’ perceptions of destinations are generally conceptualized as multidimensional constructs, encompassing attraction attributes, situational experience, cultural resources, social interaction, as well as service and infrastructural conditions [55–58]. Although variations exist in the terminology and categorization of these dimensions across different studies, they largely converge around these core elements. Within the context of agricultural heritage landscapes, the present study contextualizes these theoretical dimensions by operationalizing them into five thematic categories: “Terraced Landscape,” “Season/Time/Climate,” “Culture and Heritage,” “Human Factors,” and “Services,” thereby establishing a structural correspondence between the thematic classification and the existing theoretical framework.

#### 3.3.2. Semantic co-occurrence network analysis.

To further explore the relationships between words, this study conducted a semantic co-occurrence network analysis. When constructing co-occurrence networks, common similarity or distance metrics include the Jaccard Coefficient, Cosine Similarity, and Euclidean Distance [59]. Given that the UGC texts analyzed in this study vary substantially in length and that words may appear multiple times within a single review, the Jaccard Coefficient and Euclidean Distance—both of which are sensitive to text length and absolute word frequency—are less effective in capturing meaningful co-occurrence relationships between terms [60,61]. In contrast, Cosine Similarity applies vector normalization, which effectively mitigates the influence of text length differences and is therefore more suitable for UGC data characterized by heterogeneous text lengths.

Beyond theoretical considerations, this study also conducted comparative tests of different similarity measures. The results indicate that both the Jaccard Coefficient and Euclidean Distance exhibit limitations when applied to the two terraced landscape datasets. In the Hani Terraces dataset, the Jaccard Coefficient is strongly affected by word frequency and text length variations, leading to the formation of fragmented and isolated clusters and an overemphasis on connections among place names. Although Euclidean Distance can partially reflect stratification within the review data, it places greater emphasis on absolute word frequencies rather than on co-occurrence patterns. In the Longji Terraces dataset, the Jaccard Coefficient generates an excessively large core cluster due to chain-like co-occurrence effects among high-frequency words, while Euclidean Distance likewise fails to clearly represent the hierarchical structure of semantic relationships. Taking into account both theoretical suitability and the results of comparative testing, Cosine Similarity was ultimately selected as the primary method for semantic co-occurrence network analysis in this study. Its mathematical definition is as follows:


$$\begin{array}{c} Co\;\sin\;e\left(A,B\right)=\frac{\sum_{i=1}^{n}a_{i}b_{i}}{\sqrt{\sum_{i=1}^{n}\overset{2}{\underset{i}{a}}}\cdot \sqrt{\sum_{i=1}^{n}\overset{2}{\underset{i}{b}}}} \end{array}$$

In the formula, a_i_ and b_i_ denote the number of times terms A and B appear in the i-th document, respectively.

During result interpretation, this study will further examine the context of words within their original texts using the method Key Word in Context (KWIC) to discern their specific semantics. Ultimately, through the integration of word frequency analysis and semantic co-occurrence network analysis, this study will explore the differences in tourists’ perceptions of the two terraced fields.

#### 3.3.3. Negative comment analysis.

Ctrip’s user ratings range from 1 to 5 points, where 4–5 points correspond to Satisfied and Very Satisfied experiences, while 3 points and below indicate Average and Dissatisfied experiences. For this study, comments rated 4–5 are uniformly defined as “positive comments”, while those rated 3 or below are classified as “negative comments”. To identify key factors driving low ratings, negative comments rated 3 or below were extracted as the analysis sample. Word frequency statistics were conducted using KH Coder 3.

It should be noted that a small number of comments exhibit inconsistencies between numerical ratings and textual sentiment. Rather than conducting fine-grained sentiment classification at the level of individual comments, this study focuses on high-frequency words and recurring themes within low-rated comments to reveal the primary sources of visitor dissatisfaction at the group level. This approach helps reduce the influence of isolated rating–text mismatches on the overall results, although such simplification should be regarded as one methodological limitation of the study.

Building on this, SPSS 27.0 was employed to conduct chi-square tests, focusing on the Adjusted Standardized Residual (ASR) values of words within negative comments. An ASR > 2 indicates that the word significantly contributes to the overall chi-square difference, signifying its prominent influence in negative evaluations. The statistical results were used to observe the distribution positions of negative word frequencies across the two terraced fields.

## 4. Results

### 4.1. Overall comment data analysis

**Fig 3:**
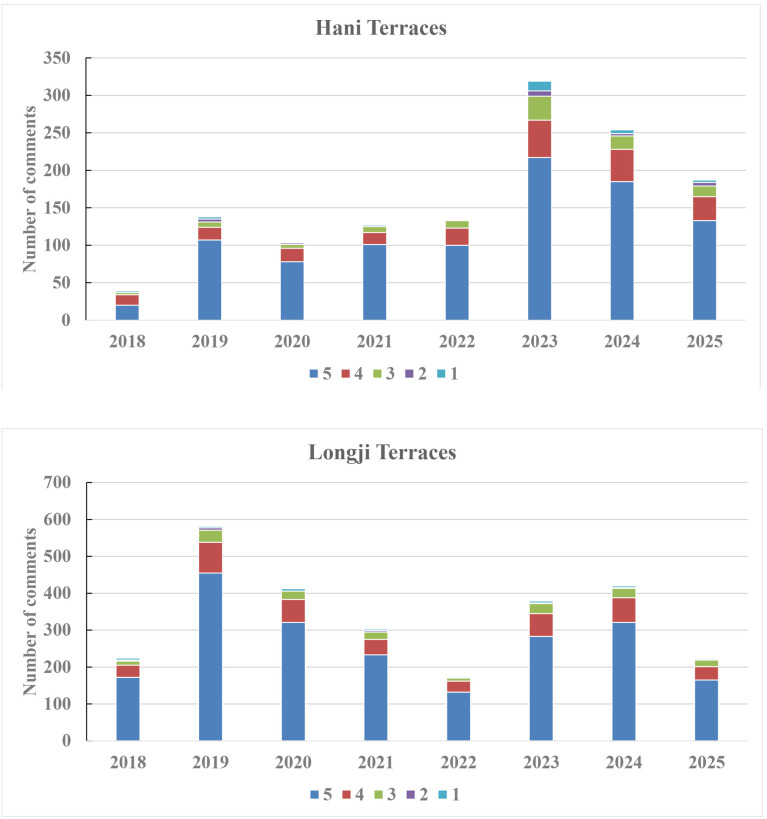
Distribution of annual comment numbers and scores.

As shown in Fig 3, this study systematically analyzed the annual distribution of comment numbers and scores for both terraced fields. Overall, the number of visitor comments for Longji Terraces exceeded that of Hani Terraces, indicating the markedly higher visibility and appeal in the tourism market. Regarding annual comment numbers, Hani Terraces maintained relatively stable comment numbers from 2019 to 2022, and it peaked in 2023 before gradually declining thereafter. In contrast, Longji Terraces reached its highest comment number in 2019 but experienced a continuous downward trend afterward. Although a slight rebound occurred in 2023, the overall scale failed to recover to 2019 levels. Considering the broader context of restricted tourism activity between 2020 and 2022, the COVID-19 pandemic likely exerted a significant influence on tourist travel behavior and the frequency of online reviews. After the pandemic, comment numbers for the Hani Terraces rebounded rapidly and peaked in 2023, which may be associated with the recovery of travel demand and an increased willingness to share experiences online. In contrast, the recovery in comment numbers for the Longji Terraces remained relatively limited, reflecting differences in review-posting behavior between visitors to the two sites. Further analysis of annual rating distributions reveals that both sites predominantly received 5-star ratings followed by 4-star ratings, indicating positive visitor experiences and consistently high overall satisfaction levels at both terraced heritage sites.

**Fig 4:**
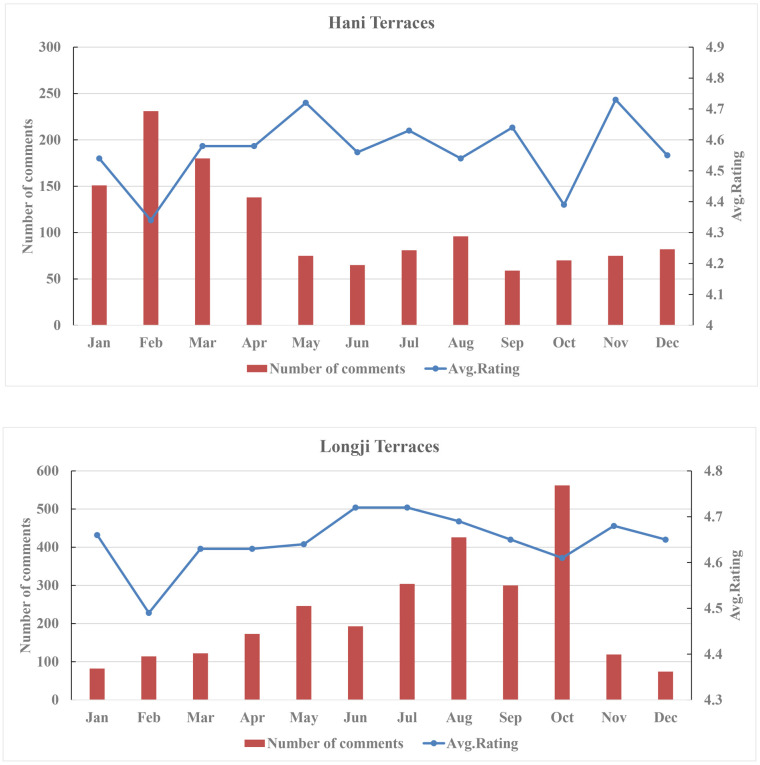
Distribution of monthly comment numbers and scores.

Fig 4 further illustrates the distribution differences in monthly comment numbers. Comments on Hani Terraces were concentrated between January and April, reaching their highest point in February. This period coincides with the terraces’ irrigation season (November to April of the following year), particularly the stable irrigation phase from December to March. During this time, the mirror-like rice paddies and the interplay of light and shadow create enhanced visual effects, forming the optimal viewing season. An examination of comment content during this period indicates a marked increase in landscape-related descriptions associated with irrigated terraces, with frequent aesthetic references to features such as “Mirror-like Paddies” and “Interplay of Light and Shadow.” Meanwhile, the Chinese New Year holiday is usually in February, leading to a substantial increase in visitor numbers and consequently driving a significant rise in comments. In contrast, comment numbers from May to October show a marked decline, remaining at relatively low levels throughout. Comments for Longji Terraces exhibit a pattern where summer and autumn months significantly outnumber those in winter and spring, peaking in October. This phenomenon is closely tied to the rice maturation and autumn harvest scenery from late September to October, during which comment content shows heightened attention to mature rice landscapes, frequently featuring color-related descriptions such as “Golden Terraces.” Combined with the National Day holiday in October, visitor numbers increase substantially, leading to a sharp increase in comments. However, from November to April of the following year, comments decline rapidly due to the diminished landscape appeal after harvest, gradually recovering only after spring seedlings turn green again.

In terms of monthly average rating distribution, both sites recorded their lowest average scores in February, followed by October. Hani Terraces saw the peak number of comments in February, yet its average rating remained the lowest throughout the year. Longji Terraces similarly exhibited a high comment number coupled with relatively low ratings in October. This outcome indicates an inverse relationship between comment number and average rating. Hani Terraces reached peak viewing value and visitor volume in February, yet their average rating hit the lowest point of the year due to overcrowding and heightened visitor expectations. Longji Terraces exhibited a similar pattern in October. This phenomenon likely correlates with visitor congestion, service capacity pressures, and unmet travel expectations.

**Fig 5:**
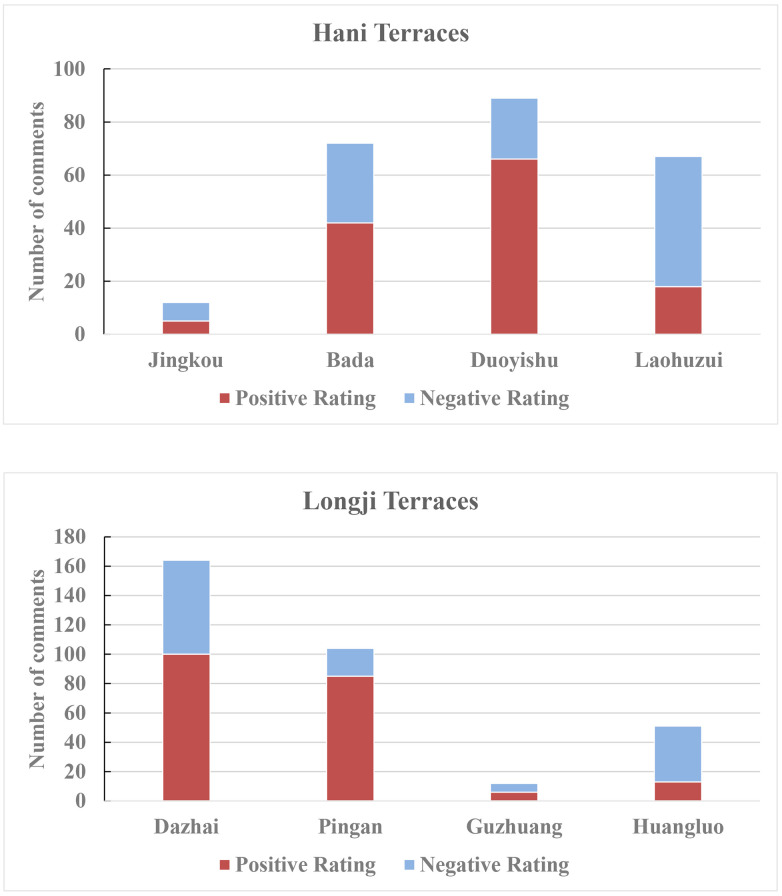
Number of comments and rating structure for each tourist attraction.

Fig 5 illustrates the differences in the number of comments and the evaluation structure among various attractions within the scenic area. In terms of total comment numbers, the numbers of reviews for Bada, Duoyishu, and Laohuzui in Hani Terraces significantly exceed that of Jingkou. This indicates that among the four main attractions, the former three hold greater advantages in visitor attention and visit frequency, while Jingkou, as a cultural village-type attraction, has relatively limited recognition and appeal. Similarly, within Longji Terraces, the number of comments for Dazhai and Ping’anzhai substantially exceeds that for Guzhuang and Huangluo, revealing a pattern of visitor concentration toward core attractions.

Regarding the ratio of positive to negative comments, Duoyishu and Bada in Hani Terraces recorded positive comment rates of 74.2% and 58.3% respectively, both exceeding negative comment proportions. This reflects the strengths of these two locations in terms of scenic appeal and tourism experience. In contrast, Laohuzui had a negative comment rate of 73.1%. KWIC analysis revealed that landslides frequently close this scenic area, causing some visitors to experience unmet expectations during their actual visit and thus tend to provide negative feedback.

In terms of Longji Terraces, Ping’anzhai received positive comments in 81.7% of cases, indicating it provides visitors with a high level of positive experience. Although Dazhai had the highest overall number of comments, negative feedback accounted for 39%—a relatively high proportion. Huangluo exhibited a situation similar to that of Hani Terraces’ Laohuzui, with negative comments reaching 74.5%. KWIC analysis reveals that negative comments primarily stem from visitor dissatisfaction with Huangluo’s Long Hair Science and Technology Museum and related folk performances, reflecting a disconnect between its cultural presentation and visitor expectations, resulting in lower appeal. Guzhuang received relatively limited comments, serving mainly as a supplementary destination for travelers with ample itinerary flexibility, and occupying a secondary position within the overall tourism system.

### 4.2. Word frequency analysis

#### 4.2.1. Terraced landscapes.

As shown in Tables 3 and 4, in comments related to “Terraces”, beyond high-frequency terms like “Terraced Fields”, “Landscape”, and “Rice”, visitors often express detailed observations from two perspectives: visual aesthetics and landscape morphology. Regarding visual aesthetics, visitors predominantly use terms like “Beautiful”, “Magnificent”, and “Picturesque” to convey their sensory impressions of the terraces’ overall scenery. Regarding structural perception, they use terms like “Slope Gradient”, “Lines”, and “Field Ridges” to convey their observations and understanding of the terraces’ details.

**Table 3:** Word frequency in reviews of Hani Terraces.

Terraced Landscape					Season/Time/Climate			Services			
Terraces	Terraced Fields			1,224	Seasons	Seasons	86	Food	Food		12
	Landscape			480		Spring	33		Rice Products		11
	Vision	Color	Blue	44		Autumn	7		Delicious		6
			Color	27		Winter	6		Restaurants		3
			Red	22		Summer	2	Accommodation	Guesthouse		51
			Colorful	22	Time	Time	232	Transportation	Roads	Roads	68
			Golden	21		Sunrise	213			Congestion	10
			Green	19		Sunset	210			Narrow	8
		Aesthetics	Beautiful	449		Irrigation	46			Rugged	6
			Magnificent	148	Holidays	Spring Festival	21			Potholes	3
			Picturesque	41		National Day	4		Parking	Parking Lots	21
			Masterpiece	34	Climate	Fog	154			Parking Fees	5
			Fairyland-like	19		Weather	134		Modes	Self-driving	18
			Unique	18		Rainy	33			Public Transportation	14
			Mirror-like	16		Sunny	26			Motorcycles	2
			Sculptural	10		Overcast	13		Transportation		37
			Vivid	5	**Culture and Heritage**			Ticketing	Tickets		195
			Palette	2	Culture	Ethnic	174	Management	Management		29
	Shape	Area		66		Culture	63		Development		3
		Elevation		16		Folk Customs	23	Facilities	Viewing Platform		167
		Slope Gradient		15		History	21		Facilities		16
		Lines		14	Heritage	World-class	103		Museums		5
		Layers		10		Heritage	89		Signage		2
		Topography		8		Heritage List	23		Toilets		3
		Field Ridges		5		Core Zone	9	Expenses	Expenses		43
	Rice			17		Conservation	9	Relevant Personnel	Staff Members		34
Villages	Villages			22		UNESCO	3		Business Owners		14
	Mushroom-shaped Houses			14	**Human Factors**				Drivers		12
	Traditional Houses			4	Local Residents	Wisdom	42		Tour Guides		4
	Buildings			3		Farmers	34				
Water Systems	Water			51		Hardworking	26				
Natural Environment	Light and Shadow			102		Simple	7				
	Mountains			95	Tourists	Tourists	42				
	Sea of Clouds			50		Photographers	37				
	Nature			45		Family Members	16				
	Land			14		Friends	12				
	Air			12							
	Sky			9							
	Forests			6							

**Table 4:** Word frequency in reviews of Longji Terraces.

Terraced Landscape					Season/Time/‌Climate			Services			
Terraces	Terraced Fields			2,114	Seasons	Seasons	216	Food	Food		172
	Landscape			1,268		Autumn	128		Delicious		56
	Vision	Color	Golden	237		Spring	47		‌Bamboo Chicken		48
			Green	139		Summer	15		Restaurants		44
			Color	13		Winter	5		Rice Products		25
			Blue	4	Time	Time	613		Fish Dishes		15
		Aesthetics	Beautiful	1,112		Irrigation	101		Cured Meat		14
			Magnificent	355		Sunrise	82		Bamboo Sh Cosine oot Dishes		12
			Picturesque	49		Sunset	36		Local specialty		7
			Mirror-like	42	Holidays	National Day	80	Accommodation	Guesthouse		286
			Fairyland-like	39		Spring Festival	12	Transportation	Roads	Roads	453
			Unique	20		Summer Vacation	11			Narrow	62
			Masterpiece	9	Climate	Fog	137			Potholes	33
			Vivid	4		Rainy	129			Rugged	24
			Sculptural	1		Climate	100			Congestion	6
	Shape	Layers		49		Sunny	31		Parking	Parking Lots	118
		Lines		36		Overcast	16			Parking Fees	52
		Elevation		27		Snowy	16		Modes	Cable Car	474
		Area		20	**Culture and Heritage**					Self-driving	67
		Slope Gradient		19	Culture	Ethnic	153			Public Transportation	41
		Field Ridges		10		Folk Customs	82			Motorcycles	5
		Topography		4		Culture	27		Transportation		63
	Rice			155		History	26	Recreation	Performance		39
Villages	Villages			44	Heritage	World-class	45		Costume		20
	Traditional Houses			20		Heritage	7	Ticketing	Tickets		269
	Buildings			10		Conservation	2	Management	Management		43
Water Systems	Water			77	**Human Factors**				Development		13
Natural Environment	Mountains			518	Local Residents	Farmers	71	Facilities	Viewing Platform		292
	Nature			108		Wisdom	58		Facilities		28
	Air			88		Simple	27		Landscape Lighting		12
	Light and Shadow			50		Hardworking	20		Signage		7
	Sea of Clouds			17	Tourists	Family Members	142		Toilets		3
	Sky			9		Tourists	105		Museums		2
	Land			7		Friends	65	Expenses	Expenses		47
	Forests			4		Disabled Person	10	Relevant Personnel	Drivers		260
						Photographers	3		Tour Guides		211
									Business Owners		88
									Staff Members		63

Further comparative analysis reveals distinct characteristics in the descriptions of color and form between the two terraced fields. In comments on Hani Terraces, the frequency of various color-related terms is relatively similar. Combined with the high frequency of the word “Area”, this indicates that visitors’ perceptions primarily focus on the visual experience of color transitions across the vast expanse of terraces. In contrast, among the comments on Longji Terraces, the term “Golden” appeared 237 times, making it the most frequent color-related word. Combined with the highest-frequency shape-related term “Layers”, this reflects visitors’ preference for the seasonal rice-growing landscape—specifically, the rolling waves of golden rice fields stacked in tier upon tier as the primary sightseeing attraction.

Beyond the terraced fields, village architecture also forms a crucial part of the visitor experience. In comments on Hani Terraces, mushroom-shaped houses are mentioned as traditional dwellings with strong regional character. These structures complement the terraces, creating an overall landscape reminiscent of an idyllic painting. Comments on Longji Terraces focus more on how new buildings disrupt the visual integrity of the landscape. Some visitors use phrases like “the houses affect the scenery” and “more and more buildings are ruining the overall landscape”, reflecting the conflict between new construction and landscape harmony.

Regarding natural environments, in comments of Hani Terraces, “Light and Shadow” appeared most frequently with 102 occurrences, indicating visitors’ preference for the terraced landscape under shifting light and shadow. In contrast, comments of Longji Terraces featured “Mountain” as the most frequent term (518), highlighting visitors’ overall impression of the terraces’ majestic, layered appearance against the mountain backdrop. Notably, the term “Forest” appeared in comments for both sites, but with low frequency. Given forests’ critical role in water conservation and ecological cycles within the terraced field systems, this low frequency suggests that casual visitors often perceive forests as merely background elements of the terraced landscape, failing to recognize their core function in ecological support and landscape maintenance.

#### 4.2.2. Season/Time/Climate.

In terms of seasons, the word frequency distribution reveals that the peak viewing season for Hani Terraces is spring, with the word “Spring” appearing 33 times, higher than any other season. Conversely, Longji Terraces saw its main tourist period in autumn, where “Autumn” (128) emerged as the most frequent term. Correspondingly, holiday visitor comments also exhibit distinct seasonal patterns. The comments on Hani Terraces exhibited the highest frequency for “Spring Festival”, indicating prominent tourism demand during this period. Conversely, comments on Longji Terraces showed the highest frequency for “National Day”, highlighting the National Day holiday in October as their hottest tourism season.

Regarding the scenic viewing period, the terms “Sunrise” and “Sunset” appeared 213 and 210 times respectively in comments of Hani Terraces, indicating a high frequency and revealing visitors’ preference for light and shadow changes and time-sensitive landscapes, with sunrise and sunset becoming the core periods. While comments about “Sunrise” and “Sunset” also appeared in comments on Longji Terraces, their overall prominence is noticeably lower than that of Hani Terraces. Regarding weather factors, “Fog” is a high-frequency term common to both sites, reflecting the widespread impact of foggy conditions on the terraces’ viewing experience.

#### 4.2.3. Culture and heritage.

In cultural and heritage commentary, visitors consistently demonstrate a high level of interest in local ethnic minorities and their folk cultures. However, perceptions of heritage attributes reveal distinct differences between the two terraced landscapes. Specifically, visitors to Hani Terraces not only view them as a scenic attraction but also explicitly recognize their status as a World Cultural Heritage site. Terms like “World-class”, “Heritage”, and “Heritage list” appear frequently in their comments, alongside heritage-specific expressions such as “Core Zone” and “UNESCO”. This indicates that Hani Terraces’ World Heritage identity has established a relatively solid cognitive framework among visitors, with the World Heritage label exerting a certain influence on their tourism experience and cultural understanding. In contrast, comments on Longji Terraces show a lower overall frequency of terms related to “heritage”. This suggests that visitors’ perceptions remain largely focused on the natural landscape and leisure tourism experience, with insufficient recognition of its potential heritage value.

#### 4.2.4. Human factors.

Visitor comments not only highlight the tangible terraced landscapes but also acknowledge the local residents who sustain and preserve them. The term “Tourists”, functioning as a label for the other, more effectively reveals the differences between the two places. For comments on Hani Terraces, the term “Photographers” ranks second only to “Tourists” in frequency, reflecting the site’s image construction as a photography destination. Correspondingly, Longji Terraces comments feature higher frequencies of familial and companion-related terms like “Family Members” and “Friends”, indicating its leisure tourism character centered on family and friend travel. Meanwhile, the presence of the term “Disabled Person” demonstrates the diversity of visitors.

#### 4.2.5. Services.

At the service level, the two destinations exhibit distinct differences. Visitors to Hani Terraces showed relatively little interest in scenic area services, with the few comments primarily focused on “Transportation” and “Ticketing”. In contrast, Longji Terraces received more service-related comments covering a broader range of aspects, including dining, transportation, entertainment, and supporting facilities, which reflects the maturity and diversity of its tourism service system.

Regarding the dining experience, terms like “Food” and “Delicious” appeared frequently in comments on Longji Terraces. Visitors provided detailed descriptions of ingredients and cuisine categories, highlighting regional specialties such as “Chicken”, “Rice”, “Fish”, and “Bamboo Shoots”. Bamboo Chicken and Bamboo Shoot Dishes are sourced from the mountains, and rice, rice noodles, and rice wine all originate from the local terraced fields. This journey from landscape to table allows visitors to not only appreciate the visual beauty of the terraced fields but also extend their sensory experience through taste, creating a multidimensional and immersive journey.

In terms of infrastructure, the terms “Roads” and “Parking” appeared frequently, indicating that poor road conditions and parking difficulties are common issues in both locations. Longji Terraces, due to their higher level of development, offer diverse sightseeing options such as cable cars, complemented by activities like performances by the Hongyao ethnic group known for their long hair, photos in ethnic costumes, and nighttime terraced field illuminations, thereby enriching visitor experiences. Regarding service personnel, terms like “Drivers”, “Tour Guides”, “Business Owners”, and “Staff Members” appeared frequently in Longji Terraces comments, reflecting visitors’ greater reliance on professional tourism services.

### 4.3. Semantic co-occurrence network

#### 4.3.1. Semantic co-occurrence network results of Hani Terraces.

**Fig 6:**
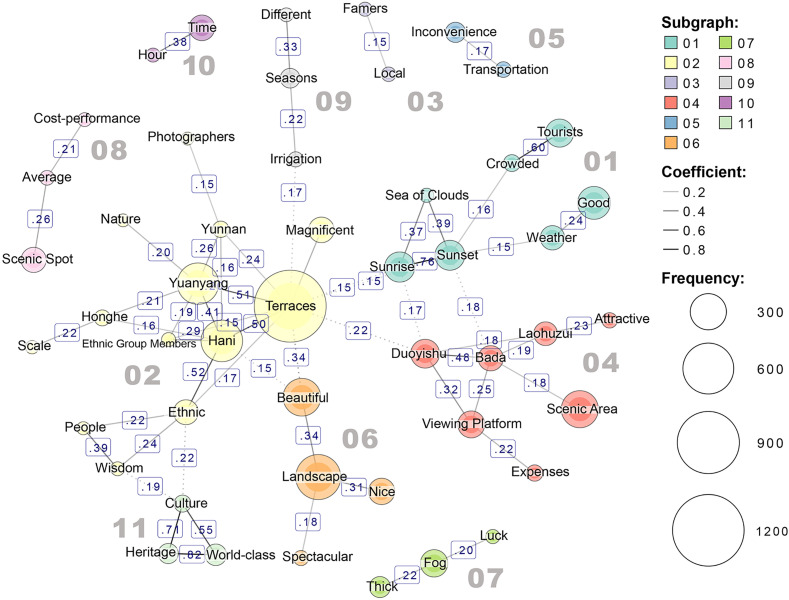
Semantic co-occurrence network results of Hani Terraces.

As shown in Fig 6, the semantic co-occurrence network of Hani Terraces reveals 11 word clusters(modularity distance). The core noun “Terraces” is located in cluster 02, which exhibits a certain degree of connection with clusters 01, 04, 06, 09, and 11, while showing no significant association with clusters 03, 05, 07, 08, and 10. Based on this, the study defines cluster 02 as the core group. Clusters 01, 04, 06, 09, and 11, which are connected to it, are designated as core supplementary clusters. Clusters 03, 05, 07, 08, and 10, which are not directly connected to it, are classified as clusters of other tourism experiences.

In the core cluster 02, “Terraces” is closely associated with geographical and ethnic terms such as “Yunnan”, “Yuanyang”, “Honghe”, “Hani”, and “Ethnic Group Members,” indicating that visitors generally possess a basic understanding of the terraced landscape’s regional context and ethnic composition. “Magnificent” and “Scale” reflect tourists’ common perception of the terraced landscape, while “Wisdom” and “People” reveal their admiration for the ingenuity of ethnic minorities in carving out these terraces. Additionally, “Photographers” indicates that tourists widely view Hani Terraces as a photography destination with high aesthetic value and visual appeal. Further KWIC analysis reveals that Hani Terraces are frequently hailed by visitors as a “photographer’s paradise”, highlighting the vital role of their visual characteristics in tourist perceptions. However, some comments, such as “Photographers are hogging all the prime spots”, reflect how photographers occupy the best vantage points on viewing platforms, limiting ordinary visitors’ sightseeing experience and reducing visibility, thereby creating a disparity in perceived experiences. Overall, the core cluster collectively embodies visitors’ foundational knowledge and the prevailing sentiment toward Hani Terraces.

Analysis of the supplementary clusters reveals that visitors’ experiences of terraced field tourism have been further expanded and refined beyond the core cluster. Cluster 01 primarily reflects temporal characteristics of sightseeing, showing a significant increase in visitor numbers during “Sunrise” and “Sunset”. Analysis on supplementary clusters employing KWIC reveals that “Sunrise” and “Sunset” frequently co-occur with “Sea of Clouds” as key objects of appreciation. Cluster 04 refers to specific scenic spots and viewing platforms, indicating visitors’ focus on particular observation points. Cluster 06 concentrates on visitors’ direct evaluations of the terraced landscape, while “Irrigation” and “Seasons” in cluster 09 reflect the distinctive scenic features and seasonal characteristics of terraced field viewing. Cluster 11 extends the perspective to cultural and heritage dimensions, indicating widespread visitor recognition of Hani Terraces as a significant World Cultural Heritage site.

Other word clusters of tourism experience reflect aspects that are not directly related to the core experience. Cluster 03 concerns visitors’ perceptions of “Local Farmers”, and corresponding KWIC analysis reveals significant polarization in these sentiments: on one hand, some visitors attribute positive traits like “Wisdom”, “Hardworking”, and “Simple” to the villagers; on the other hand, some comments mentioned irregular practices like arbitrary fees, leading to negative impressions on the villagers. Cluster 05 primarily reflects the common tourist experience of inconvenient transportation. Cluster 07 mainly highlights the impact of weather conditions on sightseeing experiences, particularly the uncertainty brought by “Fog”, which tourists often attribute to bad luck. Corresponding KWIC analysis shows polarized perceptions of fog: some comments highlight how fog enhances the terraced fields’ mystique and vitality, elevating their aesthetic appeal; others criticize dense fog for severely obstructing views and diminishing the overall sightseeing experience. Cluster 08 centers on visitors’ subjective assessments of the “Cost-performance”, with related comments generally reflecting dissatisfaction with the gap between travel costs and actual experiences. Cluster 10 predominantly uses objective descriptive terms like “Travel Duration”, indicating visitors’ concern over the time cost of their itineraries.

#### 4.3.2. Semantic co-occurrence network results of Longji Terraces.

**Fig 7:**
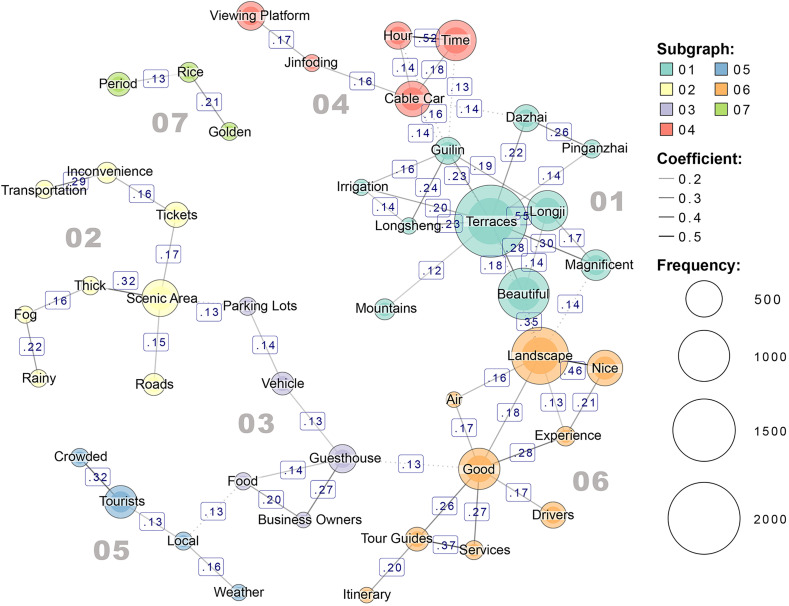
Semantic co-occurrence network results of Longji Terraces.

As shown in Fig 7, the semantic co-occurrence network of Longji Terraces can be divided into 7 word clusters(modularity distance). The core noun “Terraces” is in cluster 01, which maintains varying degrees of connection with clusters 02, 03, 04, 05, and 06. Only cluster 07 remains isolated, forming no links with other clusters. Based on this, the study defines cluster 01 as the core cluster, and clusters 02, 03, 04, 05, and 06, which are connected to it, are designated as supplementary clusters. Cluster 07, which is not directly connected to the core cluster, is classified as the cluster of other tourism experiences.

In the core cluster 01, “Terraces” co-occurs closely with location-related terms such as “Guilin”, “Longji”, “Longsheng”, “Dazhai”, and “Ping’anzhai”, while also co-occurring with positive adjectives like “Beautiful” and “Magnificent”. This indicates that tourists generally recognize the spatial characteristics of the terraced fields and hold positive perceptions and high regard for their overall landscape.

The supplementary cluster further reflects visitors’ refined focus on their tourism experiences. Cluster 04 focuses on specific viewing platforms and transportation methods, with the “Jinfoding” viewing platform and the “Cable Car” indicating visitor interest in access routes and transport options. Terms like “Hour” and “Time” within this group suggest that tour duration and travel time constitute significant components of the visitor experience. KWIC analysis reveals visitors’ frequent mentioning of travel times to various attractions and cable car duration, underscoring the importance of time consumption in overall visitor perception. Cluster 06 primarily reflects direct evaluations of scenic beauty and environmental quality: terms like “Landscape”, “Nice”, “Experience”, and “Good” indicate sensory pleasure and positive experiences during sightseeing. Within cluster 06, “Good” is associated with terms like “Tour Guides” and “Drivers”, while cluster 03 focuses on “Guesthouse”, “Business Owners”, and “Food”, indicating that tourism service elements—including tour guide services, transportation transfers, accommodation conditions, and dining experiences—are crucial factors in visitor satisfaction. Cluster 05 reflects visitors’ perceptions of crowd density, with terms like “Tourists” and “Crowded” highlighting congestion issues during peak periods. Cluster 02 involves travel-related terms like “Transportation”, “Inconvenience”, and “Roads”, and its KWIC analysis indicates widespread visitor dissatisfaction with transportation inconvenience. Additionally, weather conditions such as “Fog” and “Rainy” significantly impact visitor experiences within scenic areas.

Cluster 07, as the cluster of other tourism experiences, did not form a close connection with the core semantic cluster. Its KWIC results indicate that this cluster primarily involves terms such as “Rice” and “Golden”, with its semantic focus leaning more toward the optimal viewing season for terraced fields. This suggests that the scenery during the peak viewing season likely stands out distinctly in visitors’ perceptions compared to the general scenery observed during other seasons.

#### 4.3.3. Comparative analysis.

From the overall co-occurrence distribution characteristics, the core clusters in the analysis of both sites are concentrated on expressions related to geographical location, foundational knowledge, and overall impressions. The supplementary clusters primarily focus on evaluations of specific scenic spots and landscapes. This indicates that visitors from both sites share consistent overall perceptions, commonly using “Beautiful” as a positive descriptor for the terraced fields. Additionally, supplementary clusters of both sites include expressions like “Tourists” and “Crowded”, revealing significant visitor concern regarding tourist volume and the sense of congestion at the scenic area.

Additionally, the two sites exhibit significant differences in semantic co-occurrence features, primarily reflected in the number and content structure of word clusters. Hani Terraces feature 11 word clusters, significantly more than the 7 clusters of Longji Terraces, indicating greater thematic diversity in their commentary content, whereas Longji Terraces’ comments are relatively concentrated and convergent. Notably, Longji Terraces’ semantic network features service-related expressions absent in Hani Terraces, revealing divergent visitor perception structures. Regarding co-occurrence content, Hani Terraces’ supplementary clusters encompass richer landscape elements, while Longji Terraces’ supplementary clusters focus more on transportation and services within the scenic area. Specifically, Hani Terraces not only feature specific place names within supplementary cluster 04 but also include humanistic elements, viewing subjects, and optimal viewing times within clusters 01 and 11. This reflects visitors’ perceptions of the terraces’ cultural value and key viewing elements—a characteristic not prominently evident in the analysis of Longji Terraces.

Regarding service-related content, service terms associated with Longji Terraces predominantly cluster into supplementary clusters 03 and 06, maintaining close ties with the core cluster. This indicates that tourism services constitute a significant component of visitors’ perceptions of the site. In contrast, service-related terms for Hani Terraces are scattered across other perception clusters in limited numbers, making it difficult to directly regard them as a key component of the terraces’ tourism evaluation.

### 4.4. Analysis of negative comments

As shown in Tables 5 and 6, the distribution of negative comments differs between the two terraced fields. Regarding the terraced landscape, since the term “terraced fields” itself is a keyword, its frequent appearance in negative comments does not directly indicate negative sentiment and thus warrants no further discussion here. Among the comments on Hani Terraces, the ASR for “Landscape” is relatively high. KWIC analysis reveals that negative comments related to “Landscape” can be categorized into two types: the first involves tourists highly praising the terraced landscape but assigning lower ratings due to other negative factors like transportation or service quality, and the second category directly addresses the landscape itself, failing to meet expectations, often related to visiting outside the optimal season. Tourists express disappointment at missing the terraced fields’ mirror-like effect during the prime viewing periods. In contrast, the ASR of “Landscape” for Longji Terraces is only 0.3, showing no significant negative impact. Additionally, within the terraced landscape system, “Buildings” also exerts a certain influence. Hani Terraces experienced a decline in visitor experience due to the renovation of buildings in some villages, while Longji Terraces saw the overall landscape harmony diminished by the mix of old and new structures.

**Table 5:** ASR of negative comments on Hani Terraces.

Terraced Landscape					Season/Time/‌Climate			Services			
Terraces	Terraced Fields			2.1	Seasons	Seasons	0.8	Food	Food		−2.8
	Landscape			6.9		Spring	−2.1		Rice Products		−2.7
	Vision	Color	Blue	0.4		Autumn	−0.9		Delicious		−2.0
			Color	1.3		Winter	0.4		Restaurants		−1.4
			Red	−0.2		Summer	−0.5	Accommodation	‌Guesthouse		−1.8
			Colorful	−1.1	Time	Time	−0.3	Transportation	Roads	Roads	4.2
			Golden	−1.1		Sunrise	−0.6			Congestion	0.7
			Green	1.9		Sunset	0.4			Narrow	0.6
		Aesthetics	Beautiful	−5.1		Irrigation	−1.8			Rugged	0.5
			Magnificent	−1.9	Holidays	Spring Festival	2.2			Potholes	2.2
			Picturesque	−1.6		National Day	−0.6		Parking	Parking Lots	5.8
			Masterpiece	−1.4	Climate	Fog	2.0			Parking Fees	1.9
			Fairyland-like	−1.1		Weather	2.7		Modes	Self-driving	−1.5
			Unique	−1.0		Rainy	−2.3			Public Transportation	2.5
			Mirror-like	−1.0		Sunny	−1.3			Motorcycles	−1.1
			Sculptural	−0.8		Overcast	−1.3		Transportation		−1.6
			Vivid	−0.5	**Culture and Heritage**			Ticketing	Tickets		0.0
			Palette	−0.3	Culture	Ethnic	−1.7	Management	Management		2.9
	Shape	Area		−2.0		Culture	−0.3		Development		−0.2
		Elevation		−1.0		Folk Customs	0.8	Facilities	Viewing Platform		−4.6
		Slope Gradient		−0.9		History	−0.7		Facilities		−0.2
		Lines		−0.9	Heritage	World-class	1.4		Museums		−1.8
		Layers		−0.8		Heritage	1.7		Signage		1.8
		Topography		−0.7		Heritage List	−0.7		Toilets		−1.4
		Field Ridges		−0.5		Core Zone	−0.4	Expenses	Expenses		3.1
	Rice			−1.0		Conservation	−0.4	Relevant Personnel	Staff Members		2.4
Villages	Villages			−1.0		UNESCO	−0.3		Business Owners		−2.5
	Mushroom-shaped Houses			−0.9	**Human Factors**				Drivers		−2.2
	Traditional Houses			−0.5	Local Residents	Wisdom	−2.7		Tour Guides		−0.6
	Buildings			4.6		Farmers	0.3				
Water Systems	Water			1.3		Hardworking	−2.0				
Natural Environment	Light and Shadow			−0.7		Simple	−1.0				
	Mountains			−0.1	Tourists	Tourists	4.3				
	Sea of Clouds			0.1		Photographers	0.3				
	Nature			−1.6		Family Members	0.1				
	Land			0.3		Friends	−0.4				
	Air			0.4							
	Sky			0.7							
	Forests			−0.8							

Positive values: Observed Count > Expected Count; Negative values: Observed Count < Expected Count; Gray area: ASR > 2, indicating significant impact.

**Table 6:** ASR of negative comments on Longji Terraces.

Terraced Landscape					Season/Time/‌Climate			Services			
Terraces	Terraced Fields			6.8	Seasons	Seasons	−2.5	Food	Food		−4.3
	Landscape			0.3		Autumn	−3.3		Delicious		−4.1
	Vision	Color	Golden	−1.6		Spring	−2.1		‌Bamboo Chicken		−0.7
			Green	0.3		Summer	−1.5		Restaurants		−0.4
			Color	0.4		Winter	−0.8		Rice Products		−1.8
			Blue	−0.5	Time	Time	11.4		Fish Dishes		−1.5
		Aesthetics	Beautiful	−4.1		Irrigation	−2.0		Cured Meat		−0.8
			Magnificent	−2.1		Sunrise	−2.1		Bamboo Shoot Dishes		0.9
			Picturesque	−1.0		Sunset	−1.2		Local specialty		3.1
			Mirror-like	−0.1	Holidays	National Day	−1.7	Accommodation	‌Guesthouse		−4.7
			Fairyland-like	−1.5		Spring Festival	1.4	Transportation	Roads	Roads	−2.2
			Unique	−1.1		Summer Vacation	−1.2			Narrow	0.9
			Masterpiece	−0.7	Climate	Fog	−1.8			Potholes	7.3
			Vivid	−0.5		Rainy	−2.4			Rugged	−0.7
			Sculptural	−0.2		Climate	−1.0			Congestion	1.6
	Shape	Layers		−0.4		Sunny	−1.5		Parking	Parking Lots	2.5
		Lines		−1.4		Overcast	−1.3			Parking Fees	8.1
		Elevation		−1.2		Snowy	−1.5		Modes	Cable Car	−0.2
		Area		0.0	**Culture and Heritage**					Self-driving	0.2
		Slope Gradient		−1.0	Culture	Ethnic	−1.9			Public Transportation	2.9
		Field Ridges		−0.7		Folk Customs	2.8			Motorcycles	3.1
		Topography		−0.5		Culture	−0.2		Transportation		−1.6
	Rice			−0.8		History	−1.3	Recreation	Performance		2.4
Villages	Villages			2.5	Heritage	World-class	0.8		Costume		−0.8
	Traditional Houses			3.0		Heritage	−0.6	Ticketing	Tickets		5.7
	Buildings			2.1		Conservation	−0.3	Management	Management		5.9
Water Systems	Water			1.0	**Human Factors**				Development		−2.0
Natural Environment	Mountains			−2.3	Local Residents	Farmers	0.3	Facilities	Viewing Platform		−2.1
	Nature			−0.3		Wisdom	−2.5		Facilities		−0.2
	Air			−0.8		Simple	−1.0		Landscape Lighting		−0.5
	Light and Shadow			−1.7		Hardworking	−1.4		Signage		7.8
	Sea of Clouds			1.2	Tourists	Family Members	−1.3		Toilets		0.4
	Sky			−0.7		Tourists	1.8		Museums		0.9
	Land			−0.6		Friends	−0.9	Expenses	Expenses		6.8
	Forests			−0.5		Disabled Person	10.2	Relevant Personnel	Drivers		−3.2
						Photographers	−0.5		Tour Guides		−4.2
									Business Owners		−4.4
									Staff Members		7.2

Positive values: Observed Count > Expected Count; Negative values: Observed Count < Expected Count; Gray area: ASR > 2, indicating significant impact.

In terms of season, time, and climate, the “Spring Festival” at Hani Terraces exhibits a significant negative impact. Coinciding with the irrigation season, the surge in visitor numbers leads to increased pressure on viewing order, transportation, and declining service quality, triggering a concentration of negative comments. In addition, “Climate” factors, particularly “Fog”, serve as a major source of negative influence. Obstructed by clouds and mist, visitors often cannot fully appreciate the terraces’ panoramic views, creating a gap between expectations and reality that generates numerous negative comments. In contrast, at Longji Terraces, “Time” is the primary source of such negative feedback. This manifests in issues like poorly planned internal routes, traffic congestion, and the lack of appeal in Huangluo Village’s long-hair ethnic performances, leading visitors to feel their time is wasted and consequently lowering overall satisfaction. It is important to note that while “Season” is a significant variable affecting visitor viewing experiences, its ASR is not statistically significant for the terraces in either site. This indicates that although visitors recognize the impact of seasonality on viewing quality, their actual comments exhibit relatively mild negative feedback regarding this factor.

Regarding culture and heritage, visitors to Hani Terraces generally gave positive comments on their cultural value and heritage attributes, with a relatively strong sense of cultural identity. In contrast, the “Folk Customs” of Longji Terraces exhibit a negative impact. Its KWIC analysis reveals that among negative comments related to folk customs, the long-hair performance in Huangluo is the primary factor. As a significant local folk tradition, the “Long-hair Tradition” should possess high appeal. However, most visitors express disapproval of its presentation style, viewing it as a waste of time and money. This indicates that the content of the long-hair ethnic performances requires improvement and enhancement.

Regarding human factors, negative comments associated with “Tourists” are particularly prominent on Hani Terraces. This manifests primarily during peak tourist seasons when large influxes of visitors exceed the scenic area’s carrying capacity, leading to disorder and friction among tourists—key sources of negative comments. In the comments on the Longji Terraces, the ASR for the term “Disabled Person” reaches 10.2, reflecting heightened visitor expectations for accessible facilities and services catering to diverse groups.

In terms of services, transportation, and scenic area management issues constitute the primary negative factors. As shown in Tables 5 and 6, high ASR values are associated with factors such as road conditions, parking, and management. Notably, neither terraced site exhibits significant negative factors related to food and lodging. Visitors to Hani Terraces directed relatively little attention to dining and accommodation in their negative comments, resulting in fewer related complaints. Conversely, tourists at Longji Terraces generally offered positive feedback on food and lodging, with dining quality and accommodation conditions receiving consistently high evaluations across most comments. Only the “Local Specialty” factor showed some low ratings, with an ASR value of 3.1, primarily concentrated in Huangluo Village. Comments highlighted instances in which villagers aggressively promoted local products such as shampoo, which caused visitor dissatisfaction and led to negative evaluations.

## 5. Discussion

From the perspectives of destination perception and perceived value research, tourists’ perceptions and evaluations of destinations are typically characterized by differentiated emphases on the importance of various experiential elements, rather than by an averaged perception of all environmental attributes [62]. In terrace tourism research, Yang et al. found that tourists’ perceptions mainly focus on landscape, ecology, culture, and services, with landscape and services more likely to become the main focus of evaluation [46]. The findings of this study are broadly consistent with this conclusion, showing that tourist evaluations at terraced heritage sites are not evenly distributed across all types of elements, but instead exhibit clear differences in emphasis. More importantly, this study further indicates that, in the context of World Heritage terraced landscapes, such differences are not simply a matter of whether landscape or services are more important. Rather, they are shaped by the salience of heritage identity, local development models, and governance orientations, which give rise to different perceptual structures and, in turn, influence tourists’ evaluative logic and the formation of negative experiences.

More specifically, Hani Terraces exhibit a perceptual structure centered on heritage cognition. In the review texts, the frequent attention given to keywords such as “World-class”, “Heritage” and “Core Zone” together with the concentration of Group 11 in the semantic co-occurrence network, suggests that visitors are more inclined to understand the value of Hani Terraces within the cognitive framework of World Cultural Heritage. As Poria et al. have argued, tourists attach specific meanings to World Heritage designation, which in turn influences their experience of and value perception at heritage sites [63]. Similarly, Bui’s research shows that World Heritage identity, as an institutional marker, can shape visitors’ overall impressions of a place and the evaluative framework through which they interpret it [64]. The empirical findings of this study further extend these arguments. The World Heritage label not only strengthens visitors’ awareness of the heritage attributes of Hani Terraces, but also shapes the way they interpret the natural landscape. Seasonal changes, light and shadow effects, and irrigated mirror-like scenery are not perceived merely as isolated aesthetic features of nature. Rather, they are incorporated into a broader meaning system associated with cultural landscape heritage, thereby forming an overall perceptual structure centered on heritage value.

By contrast, Longji Terraces display a more evident service-embedded pattern. In the semantic co-occurrence network, service-related terms such as “Tour Guides”, “Food” and “Guesthouse” are connected with “Terraces”, indicating that when visitors describe their terrace experience, they tend to evaluate it within a concrete tourism service context. This finding echoes Yang et al.’s conclusion that service factors are particularly prominent in visitors’ perceptions of terraced agricultural heritage landscapes [46]. Building on this, the present study further suggests that even among terraced heritage sites, visitors’ understanding of place meaning is not necessarily dominated by heritage value. In Longji Terraces, the terraces are more often experienced as the main attraction within a leisure itinerary, and their meaning is largely embedded in services such as guiding, dining, and accommodation. As a result, a relatively clear service-oriented pattern emerges.

To some extent, this divergence in perceptual focus reflects the influence of different local governance and development models on the construction of tourists’ cognition. As Wang and Marafa have shown in their discussion of tourism imaginaries and power relations in Hani Terraces, the governance space of a destination and its key power actors play an important role in shaping tourists’ imaginaries of place [44]. In the case of Hani Terraces, local governments have remained deeply involved in the delineation of protected area boundaries and the maintenance of cultural landscape integrity during both the World Heritage nomination process and subsequent conservation efforts [65]. This governance background has not only strengthened the institutional expression of World Heritage identity, but has also created the conditions for visitors to understand place value within a heritage framework. By contrast, although Longji Terraces have been recognized as a Globally Important Agricultural Heritage System, tourism practice there has mainly focused on leisure and consumption-oriented activities such as terrace sightseeing, song-and-dance performances, and hospitality services [66]. The connectivity between the service cluster and the core landscape cluster in the semantic network suggests that under this development orientation, visitors are more likely to understand the terraces as part of a leisure consumption experience rather than primarily as a heritage object to be interpreted and understood.

Different governance and development models not only shape tourists’ perceptual structures, but also influence their core expectations to some extent, and this difference is further reflected in the negative evaluations found at the two sites. This study shows that visitors to Hani Terraces are relatively sensitive to natural conditions such as “fog” and “weather.” Given the World Heritage status of Hani Terraces, visitors often hold high expectations for the presentation of the landscape. When the mirror-like effect during the irrigation season is not fully visible, or when visibility is obstructed, fluctuations in natural conditions are more likely to be translated into negative evaluations. This is consistent with Wen et al.’s argument that aesthetic expectations can have a direct negative effect on tourist satisfaction [67]. By contrast, negative feedback at Longji Terraces is more often concentrated on mismatches between time costs and experiential returns. When visitors’ expectations are oriented more toward leisure efficiency and consumption experience, they are more concerned with the rationality of itinerary arrangements and the richness of activities. This also corresponds with Wang et al.’s findings from research on Japanese terraced landscapes, which showed that when visitors have high expectations regarding itinerary planning and accessibility, “bad timing” and “poor accessibility” often become important sources of negative experiences [45]. These findings suggest that the differences in negative evaluations between the two sites are not random, but instead represent specific manifestations of unmet core expectations under different perceptual structures.

When comparing heritage sites with different development and governance contexts, research should not remain limited to differences in experience themes or high-frequency elements. It should also identify how visitors organize their cognition of place and examine how such cognitive structures relate to local governance orientations and tourism development models. Among similar agricultural heritage sites, different development philosophies may produce different perceptual structures, core expectations, and mechanisms through which negative evaluations emerge. In this sense, the present study helps move terrace tourism research from a comparison of experiential content toward a comparison of evaluative logic and perceptual construction mechanisms, and provides a more explanatory analytical perspective for understanding differences in tourism experiences across heritage sites.

Although this study provides a relatively in-depth comparison of visitor experiences at the two terraced sites, certain limitations remain. First, the research exclusively utilized Ctrip as its data source, excluding user comments from other platforms. Differences in user composition and commenting tendencies across platforms may have influenced the findings. Second, UGC is inherently non-random, as reviews are often posted voluntarily by visitors with particularly strong experiences, which may amplify extreme positive or negative sentiments while underrepresenting neutral experiences. This self-selection bias may affect the observed structure of perceived visitor experiences. Third, the analysis is limited to textual content and does not incorporate visual information such as photographs and videos included in reviews. Moreover, the sample primarily represents domestic Chinese tourists and does not capture evaluations posted by international visitors on platforms such as Twitter or Google Maps. Future research could integrate multi-platform, multi-group, and multi-type data to further deepen the understanding of differences in visitor experiences.

## 6. Conclusion

This study analyzes online visitor comments on Hani Terraces and Longji Terraces, to examine overall tourism experience characteristics, visitor perception differences, and negative comment content. Three primary conclusions emerge as follows. First of all, regarding the overall tourism experience, Longji Terraces attracted more visitors due to its higher market recognition, yet its post-pandemic recovery proved relatively limited. In contrast, Hani Terraces exhibited a rapid rebound in comment volume during the post-pandemic period, reflecting differences in tourists’ online expression behaviors between the two sites. At the same time, both destinations exhibit a pattern of concentrated visitor comments during holidays and peak viewing seasons; however, these periods are associated with lower ratings, indicating tensions between scenic area management capacity and visitor satisfaction. Secondly, in terms of visitor perception differences, visitors of Hani Terraces generally demonstrate stronger recognition of heritage value, with their travel experiences leaning toward cultural and heritage appreciation. Conversely, visitors to Longji Terraces primarily emphasize tourism and leisure services, showing relatively limited awareness of its agricultural heritage value. Thirdly, regarding negative comments, common complaints at both sites centered on transportation and management issues. Additionally, weather emerged as another negative factor affecting Hani Terraces’ visitor ratings, while Longji Terraces saw concentrated dissatisfaction with tourism services.

The research findings offer valuable insights for tourism development in terraced heritage sites. In tourism promotion and experience design, it is necessary to balance the expression of heritage value and the development of service systems according to different development orientations, thereby avoiding the over-concentration of visitor expectations under a single narrative framework. At the same time, considering the observed pattern of high comment volumes but relatively low ratings during holidays and peak viewing seasons, scenic areas should adopt rational spatial layouts and route integration to relieve pressure on core attractions and promote more balanced development of non-core sites. In response to visitor dissatisfaction with the modes of folk cultural expression, it is necessary to further and more systematically explore folk cultural content associated with terraced landscapes in order to enhance its attractiveness and cognitive value. In addition, given that transportation conditions and scenic area management issues constitute major sources of negative evaluations, efforts should be made to improve tourism service systems, particularly by strengthening transportation organization and scenic area management to increase carrying capacity and visitor comfort. Through these measures, a more coordinated development between heritage conservation and tourism utilization can be achieved.

## Supporting information

S1 Appendix Synonym merging scheme for semantic analysis. (DOCX)
