# Supplementary material for: Local tourism experiences at World Heritage rice terrace sites in China: A comparative study of Hani Terraces and Longji Terraces
Source: PLoS One. 2026 Jun 1;21(6):e0349872. doi: 10.1371/journal.pone.0349872 (PMC13225647; doi:10.1371/journal.pone.0349872)
Supplement: S1 Appendix — (DOCX) [file pone.0349872.s001.docx]

**S1 Appendix. Synonym merging scheme for semantic analysis.**

**Table 1. Synonym merging scheme for semantic analysis of Hani Terraces reviews.**

| **Normalized semantic category** | **Merged synonyms** |
| --- | --- |
| **Terraced Landscape** | |
| Terraced Fields | paddy field, irrigated field, farmland, dry field, rice paddy, field plot, fertile farmland |
| Landscape | scenery, landscape, vista, scene, scenic view |
| Color | None |
| Red | rosy pink, fiery red, red, jujube red, bright red |
| Colorful | multicolored |
| Golden | golden yellow, yellow, light yellow, shimmering gold |
| Green | greenish, vivid green, fresh green |
| Beautiful | pretty, very beautiful, great beauty, more beautiful, breathtaking, aesthetic, exquisite, splendid, most beautiful, uniquely beautiful |
| Magnificent | majestic, vast and magnificent, magnificent scenery |
| Picturesque | painting, scroll painting, ink wash painting, oil painting, landscape painting, printmaking, three-dimensional artwork |
| Masterpiece | artwork, fine work |
| Fairyland-like | ethereal aura, fairyland traces |
| Unique | special |
| Mirror-like | mirror, mirror-like surface |
| Sculptural | sculpture |
| Palette | None |
| Area | None |
| Elevation | None |
| Slope Gradient | slope |
| Lines | curve |
| Layers | depth, layered |
| Topography | None |
| Field Ridges | None |
| Rice | rice grain, rice ear, rice flower, rice seedling, upland rice |
| Villages | None |
| Traditional Houses | None |
| Buildings | building |
| Water | water source, water system, water surface, moisture, canal water ripples, water droplets |
| Light and Shadow | sunlight, light, glow, illumination, rosy light, daylight, light perception, light spots, soft light, water reflections, rippling light, morning light, golden light, Tyndall effect |
| Mountains | on the mountain, mountain terrain, great mountains, mountain valley, high mountains, mountain mass, mountain ranges, mountain peak, mountains and rivers, hilltop, mountain ridges, mountain ridge, deep mountains, barren mountains, distant mountains, green mountains |
| Sea of Clouds | None |
| Nature | natural world |
| Land | red soil |
| Air | None |
| Sky | None |
| Forests | None |
| **Season/Time/‌Climate** | |
| Seasons | season |
| Spring | spring atmosphere, spring and winter |
| Autumn | autumn day, autumn atmosphere |
| Winter | winter and spring |
| Summer | None |
| Time | moment, time period, time point, hour |
| Irrigation | water storage |
| Fog | cloud and mist, mist, dense fog, morning mist, haze, light mist, misty, in the fog, thick fog |
| Climate | None |
| Rainy | rainy season, rainy weather, rainy day, light rain, overcast rain, spring rain, drizzling rain, rainfall, in the rain, fine rain, misty rain, after rain |
| Sunny | sunny day, very sunny day, clear weather |
| Overcast | cloudy day |
| **Culture and Heritage** | |
| Ethnic | Hani ethnic group, Hani people |
| Culture | None |
| Folk Customs | long-table banquet, market |
| History | history of civilization, historical records |
| UNESCO | None |
| Heritage | heritage site |
| Heritage List | None |
| Core Zone | None |
| Conservation | None |
| World-class | None |
| **Human Factors** | |
| Wisdom | None |
| Farmers | mountain residents, residents |
| Photographers | photography enthusiasts, photographers |
| Friends | boyfriend |
| Family Members | father, parents, mother, husband, elderly, child |
| **Services** | |
| Food | meals, farmhouse cuisine, local cuisine, free-range chicken, dishes, food and drinks, lunch and dinner, dinner, breakfast |
| Rice Products | rice, rice noodles, brown rice |
| Delicious | tasty |
| Restaurants | restaurant |
| Guesthouse | hotel |
| Roads | road, mountain road, concrete road, main road, path, winding road, curve, asphalt road |
| Congestion | traffic relief, traffic jam |
| Narrow | narrow road |
| Potholes | large pothole |
| Parking Lots | parking space |
| Parking Fees | None |
| Self-driving | self-driving tour, self-driving car |
| Public Transportation | bus, coach, shuttle bus |
| Motorcycles | None |
| Transportation | None |
| Tickets | entrance ticket |
| Management | None |
| Development | None |
| Viewing Platform | None |
| Facilities | None |
| Museums | None |
| Signage | road sign |
| Toilets | restroom |
| Expenses | charged, free |
| Staff Members | ticket checker, service staff |
| Business Owners | female business owner |
| Drivers | driver |
| Tour Guides | None |

Note: “None” indicates that no synonymous expressions were identified; therefore, the normalized semantic category was directly used for keyword retrieval. The synonym merging scheme was originally developed based on Chinese-language review data, and the terms listed here are presented in English for publication purposes.

**Table2. Synonym merging scheme for semantic analysis of Longji Terraces reviews.**

| **Normalized semantic category** | **Merged synonyms** |
| --- | --- |
| **Terraced Landscape** | |
| Terraced Fields | rice field, irrigated field, farmland, dry field, field plot, fertile farmland, terraced field system, fields, irrigated terraces, well-irrigated field, wheat field |
| Landscape | scenery, vista, scene, scenic view |
| Golden | golden yellow, wheat-colored, bright yellow, radiant yellow, amber yellow, yellowish, yellow, golden hue, shimmering gold, golden color |
| Green | greenness, yellow-green, emerald green, green space, green waves, green rice, green sprouts, lush green, bluish green, yellowish green, chartreuse, vivid green, jade green, greenish, green seedlings, green turf |
| Blue | bluish tone |
| Beautiful | pretty, most beautiful, appeal, very beautiful, splendid, scenic, more beautiful, extremely beautiful, great beauty, marvelous, graceful, breathtaking, wonderful, really beautiful, delightful |
| Magnificent | majestic, imposing, vast and magnificent, grand |
| Picturesque | scroll painting, abstract painting, pictorial scene, landscape painting, ink wash painting, oil painting, monumental painting, pictorial conception |
| Mirror-like | mirror, mirror-like surface |
| Fairyland-like | celestial palace, ethereal aura |
| Unique | None |
| Masterpiece | artwork |
| Vivid | vivid |
| Sculptural | None |
| Layers | sense of depth, layered, multilayered, overlapping, tiered |
| Lines | curve |
| Elevation | None |
| Area | None |
| Slope Gradient | steep slope |
| Field Ridges | None |
| Topography | None |
| Rice | rice grain, rice, rice ear, rice flower, rice seedling, japonica rice, late-season rice, rice leaf, rice root |
| Villages | village, hamlet |
| Buildings | building |
| Traditional Houses | traditional dwelling, house |
| Water | stream, spring water, pond, water surface, waterside, mountain spring |
| Mountains | great mountains, between mountains, foot of the mountain, mountain ranges, high mountains, hills, mountain terrain, mountain peak, mountain summit, mountain valley, green mountains, mountain ridges, hilltop, towering mountain ranges, in the mountains, mountain land, mountain forest, mountain ridge, back mountain, mountain mass, mountain range, deep mountains, behind the mountain, mountain hill, cliff, base of the mountain, mountain hollow, mountainside, barren mountains, distant mountains, Longji Mountains, layered green mountains, mountain top, on the mountain |
| Nature | natural world |
| Air | None |
| Light and Shadow | sunlight, light, daylight, water reflections, glow, morning light, rosy light, golden light |
| Sea of Clouds | None |
| Sky | None |
| Land | None |
| Forests | woodland, forest |
| **Season/Time/‌Climate** | |
| Seasons | season |
| Autumn | golden autumn, autumn and winter, late autumn, autumn day |
| Spring | spring and autumn, spring and summer, spring atmosphere |
| Summer | summer day, midsummer |
| Winter | winter day, midwinter |
| Time | time point, time period |
| Sunrise | None |
| Spring Festival | None |
| Summer Vacation | None |
| Fog | cloud and mist, mist, dense fog, haze, light mist, foggy weather, foggy, mountain mist, morning mist, water mist |
| Rainy | rainy day, light rain, rainy weather, after rain, rainy season, heavy rain, drizzling rain, rainy and overcast, rainwater, rain mist, wind and rain, rainstorm, fine rain, drizzle, shower, misty rain, in the rain, rain |
| Climate | None |
| Sunny | sunny day, clear weather |
| Overcast | cloudy day |
| Snowy | snowflakes, heavy snow, snow cover, winter snow, white snow |
| **Culture and Heritage** | |
| Ethnic | Zhuang ethnic group, ethnic group |
| Folk Customs | None |
| Culture | None |
| History | None |
| World-class | None |
| Heritage | None |
| Conservation | None |
| **Human Factors** | |
| Farmers | residents, locals, local people |
| Wisdom | None |
| Hardworking | None |
| Family Members | whole family, father, parents, dad, mom, elderly people, husband, wife, mother-in-law, elderly, children, child |
| Tourists | tour group |
| Friends | girlfriend, boyfriend |
| Disabled Person | None |
| Photographers | photographer |
| **Services** | |
| Food | meals, farmhouse cuisine, local cuisine, dishes, dinner, breakfast, lunch, set meal, Chinese cuisine, group meal, home-style dishes, local specialties, cuisine, meal |
| Delicious | really tasty, tasty |
| Bamboo Chicken | None |
| Restaurants | small restaurant, restaurant |
| Rice Products | rice noodles, rice, rice vermicelli, rice wine, glutinous rice wine |
| Fish Dishes | fish, fish with pickled vegetables |
| Cured Meat | None |
| Bamboo Shoot Dishes | stir-fried fresh bamboo shoots, grilled bamboo shoots, dried bamboo shoots, stir-fried bamboo shoots, stir-fried sour bamboo shoots, high-mountain bamboo shoots, fresh bamboo shoots, mountain bamboo shoots, bamboo shoots |
| Local specialty | specialty products, local specialties |
| Guesthouse | large hotel, hotel, mountain guesthouse |
| Roads | road, mountain road, concrete road, main road, path, winding road, curve, mountain path |
| Narrow | narrow road |
| Potholes | large pothole |
| Congestion | traffic jam |
| Parking Lots | parking space |
| Parking Fees | None |
| Cable Car | cableway |
| Self-driving | self-driving tour, self-driving car |
| Public Transportation | bus, coach, public bus, shuttle bus |
| Motorcycles | motorcycle |
| Transportation | None |
| Performance | None |
| Costume | None |
| Tickets | entrance ticket |
| Management | None |
| Development | None |
| Viewing Platform | viewing area |
| Facilities | None |
| Landscape Lighting | None |
| Signage | road sign |
| Toilets | restroom |
| Museums | None |
| Expenses | charged, free |
| Drivers | driver |
| Tour Guides | None |
| Business Owners | female business owner |
| Staff Members | ticket clerk, service staff |

Note: “None” indicates that no synonymous expressions were identified; therefore, the normalized semantic category was directly used for keyword retrieval. The synonym merging scheme was originally developed based on Chinese-language review data, and the terms listed here are presented in English for publication purposes.
